# Combined Impact of Known Lifestyle Factors on Total and Cause-Specific Mortality among Chinese Men: A Prospective Cohort Study

**DOI:** 10.1038/s41598-017-05079-5

**Published:** 2017-07-13

**Authors:** Qing-Li Zhang, Long-Gang Zhao, Wei Zhang, Hong-Lan Li, Jing Gao, Li-Hua Han, Wei Zheng, Xiao-Ou Shu, Yong-Bing Xiang

**Affiliations:** 1 SKLORG & Department of Epidemiology, Shanghai Cancer Institute, Renji Hospital, Shanghai Jiaotong University School of Medicine, Shanghai, 200032 China; 2 Division of Epidemiology, Department of Medicine, Vanderbilt Epidemiology Center, Vanderbilt-Ingram Cancer Center, Vanderbilt University School of Medicine, Nashville, USA

## Abstract

Impact of combined lifestyles on risk of mortality needs to be explored quantitatively. We aimed to evaluate the associations of combined lifestyle factors with total and cause-specific mortality in Chinese men. We used data from the Shanghai Men’s Health Study (2002–2013), an on-going population-based prospective cohort study of men (aged 40 to 74 years). Four traditional unfavorable lifestyle factors were included: smoking, heavy alcohol use, unhealthy diet and physical inactivity. Cox proportional hazards models were used to calculate the hazard ratios (HRs) and 95% confidence intervals (CIs). Among about 61,480 men in the cohort, a total of 4,952 men died, of which 1,637 men died from cardiovascular diseases (CVD), 2,122 from cancer during a median of 9.29 years’ follow-up. The HRs of men with four risk practices comparing to those with zero were 2.92 (95%CI: 2.53, 3.38) for all-cause mortality, 3.15 (95%CI: 2.44, 4.05) for CVD mortality, and 3.18 (95%CI: 2.55, 3.97) for cancer mortality. The population attributable risks (PARs) were 0.41, 0.40 and 0.38 for total, CVD and cancer mortality, accordingly. As combined unhealthy lifestyle behaviors had substantial impact on total and cause-specific mortality, promotion of healthy lifestyle should be a public health priority.

## Introduction

Non-communicable diseases (NCDs) kill 38 million people each year and are responsible for almost 70% of deaths worldwide^1^. Statistics of world health organization (WHO) suggested that smoking, heavy alcohol use, physical inactivity and excess salt/sodium intake attributed to 6 million, 3.3 million, 3.2 million and 1.7 million annual deaths accordingly across the world^1–3^.

As individual lifestyle factors tend to cluster within a population and interaction effects of them may exist^4, 5^, emerging studies on the association of combined impact of lifestyle factors with mortality have been conducted recently. Such association would be more informative for policy making and diseases prevention.

Previous studies were mostly conducted in western countries^6–16^, while few were in Asian countries^17–21^. In addition, the definition and number of individual lifestyle factors varied between studies, which led to substantial heterogeneity of the results (I-squared = 94%)^22^.

Population in the Shanghai Men’s Health Study (SMHS) has its unique lifestyle pattern compared with their counterparts in western countries such as high percentage of smoking and low participation rate of physical activity^23–25^. Thus it is essential to explore the association and estimate the PAR in this population. In addition, results from the SMHS would also be of great significance for men in other cities of China and for men in other Asian countries with similar ethnicity, cultural factors and life style. We defined the healthy lifestyle factors according to public health recommendations scientifically and objectively. The chosen risk lifestyle factors–tobacco use, heavy use of alcohol, unhealthy diet and physical inactivity–are four modifiable lifestyle risk factors proposed by WHO that increase the risk of NCDs^1^.

Based on data from the SMHS, a prospective cohort in China, the present study aimed to investigate associations of lifestyle index with all-cause and cause-specific mortality risks and further quantify population attributable risks of these lifestyle factors. In addition, we also evaluated the mortality risk for commonly occurring combinations of these lifestyle factors.

## Results

### Descriptive Statistics

A total of 59,747 men aged 40 to 74 years at baseline were included in the current analysis. After a median follow-up of 9.29 years (a total of 550,461 person-years), 4,952 deaths occurred, of which 1,637 men died from CVD and 2,122 men from cancer. Table 1 shows the definition and distribution of individual lifestyle behaviors at risk. At baseline, 66% participants were current smokers or smoking cessation less than 10 years; 13% of participants consumed alcohol more than 14 drinks a week; 40% of participants were categorized as having unhealthy diet; and up to 83% of participants involved moderate-to-vigorous-intensity physical activity less than 150 minutes per week.

**Table 1:** Scoring of factors in the lifestyle risk index, the Shanghai Men’s Health Study (2002–2013, n = 59,747).

Health Behavior	Scoring Method (1 = At Risk)	Percentage “At Risk”
Smoking	1 = current smoker OR quitting smoking < 10 years	66%
Alcohol use	1 = consuming > 14 drinks a week	13%
Dietary behavior	1 = Chinese food pagoda score distributed in the bottom two quintile in the SMHS	40%
Physical inactivity	1 = engaging in < 2 MET-hours/day (approximately equivalent to < 150 min/week) of moderate-to-vigorous-intensity physical activity	83%

The percentage of participants with zero, one, two, three, four unhealthy lifestyle-related factors were 6.07%, 23.56%, 38.70%, 25.13% and 6.54%, accordingly. Compared to men with low lifestyle index, those with higher index tended to be younger, less educated, with lower income, more likely to be manual workers and with lower prevalence of hypertension, diabetes mellitus, coronary heart disease, and stroke (Table 2).

**Table 2:** Baseline characteristics of men by lifestyle risk index, the Shanghai Men’s Health Study (2002–2013, n = 59,747).

Sample	lifestyle score				
	0	1	2	3	4
Subject (N)	3,626	14,077	23,125	15,012	3,907
Age (years), mean (SD)^a^	65.02 (8.04)	58.83 (9.72)	54.05 (9.26)	52.67 (8.75)	51.75 (8.20)
Education, N (%)^b^					
None/elementary	336 (9)	1,031 (7)	1,297 (6)	1,012 (7)	307 (8)
Junior high school	850 (23)	3,453 (25)	7,673 (33)	6,164 (41)	1,860 (48)
High school	1,004 (28)	4,335 (31)	8,956 (39)	5,840 (39)	1,459 (37)
College and above	1,436 (40)	5,258 (37)	5,199 (22)	1,996 (13)	281 (7)
Income/person, N (%)^b^					
< 500 CNY	165 (5)	905 (6)	2,690 (12)	2,791 (19)	923 (24)
500–999 CNY	1,573 (43)	5,588 (40)	9,805 (42)	6,791 (45)	1,736 (44)
1,000–1,999 CNY	1,488 (41)	5,705 (41)	8,326 (36)	4,430 (30)	1,031 (26)
> = 2000 CNY	400 (11)	1,879 (13)	2,304 (10)	1,000 (7)	217 (6)
Occupation, N (%)^b^					
Professional	1,624 (45)	5,418 (38)	5,723 (25)	2,498 (17)	450 (12)
Clerical	798 (22)	2,957 (21)	5,156 (22)	3,339 (22)	869 (22)
Manual workers	1,204 (33)	5,702 (40)	12,246 (53)	9,175 (61)	2,588 (66)
BMI (Kg/m^2^), N (%)^b^					
< = 18.5	88 (2)	395 (3)	1,035 (4)	813 (5)	200 (5)
18.5–24	1,723 (48)	6,655 (47)	1,1601 (50)	7,846 (52)	2,084 (53)
24–28	1,497 (41)	5,828 (41)	8,636 (37)	5,148 (34)	1,292 (33)
> 28	318 (9)	1,199 (9)	1,853 (8)	1,205 (8)	331 (8)
Hypertension, N (%)^b^	1,687 (47)	5,263 (37)	6,265 (27)	3,527 (23)	1,040 (27)
Diabetes mellitus, N (%)^b^	346 (9)	1,050 (7)	1,350 (6)	811 (5)	175 (4)
Coronary heart disease, N (%)^b^	472 (13)	966 (6)	970 (4)	510 (3)	113 (3)
Stroke, N (%)^b^	263 (7)	660 (5)	774 (3)	421 (3)	115 (3)

N: number;

SD: standard deviation;

a: p-value from ANOVA test;

b: p-value from Chi-square test;

CNY: China Yuan;

BMI: Body mass index, the cut-offs were defined according to the guideline of working group on obesity in China.

All P values across all lifeatyle factors < 0.001

### Impact of Individual Risk Factor

All these four lifestyle-related factors included in the index score were independently related to all-cause and cause-specific mortality (Table 3). For all-cause mortality, smoking (HR = 1.39, 95% CI: 1.30, 1.47) and heavy alcohol use (HR = 1.30, 95% CI: 1.20, 1.40) showed the greatest impact, followed by unhealthy diet (HR = 1.25, 95% CI: 1.18, 1.33) and physical inactivity (HR = 1.20, 95% CI: 1.12, 1.28). Moreover, these lifestyle factors were all positively related to CVD and cancer mortality. For estimation of PAR of all-cause mortality, 19% of deaths in the total population was attributed to smoking (PAR = 0.19, 95% CI: 0.15, 0.23), and the following were physical inactivity (PAR = 0.13, 95% CI: 0.09, 0.18), unhealthy diet (PAR = 0.11, 95% CI: 0.08, 0.15) and heavy alcohol use (PAR = 0.05, 95% CI: 0.03, 0.06).

**Table 3:** Hazard ratios of all-cause, cardiovascular, and cancer mortality in relation to individual lifestyle factors, the Shanghai Men’s Health Study (2002–2013, n = 59,747).

Lifestyle factor		Total (n = 4952 deaths)		CVD (n = 1637 deaths)		Cancer (n = 2122 deaths)		Adjusted PAR for total mortality
Cohort N		Deaths	HR (95%CI)	Deaths	HR (95% CI)	Deaths	HR (95% CI)	PAR (95% CI)
Smoke								
0	20,231	1,902	1.00 (reference)	684	1.00 (reference)	697	1.00 (reference)	0.19 (0.15, 0.23)
1	39,516	3,050	1.39 (1.30, 1.47)	953	1.35 (1.21, 1.50)	1,425	1.63 (1.48, 1.80)	
Alcohol use								
0	51,793	4,119	1.00 (reference)	1,376	1.00 (reference)	1,730	1.00 (reference)	0.05 (0.03, 0.06)
1	7,954	833	1.30 (1.20, 1.40)	261	1.24 (1.08, 1.42)	392	1.41 (1.26, 1.58)	
Dietary behavior								
0	35,849	2,565	1.00 (reference)	838	1.00 (reference)	1,131	1.00 (reference)	0.11 (0.08, 0.15)
1	23898	2,387	1.25 (1.18, 1.33)	799	1.32 (1.20, 1.46)	991	1.16 (1.06, 1.27)	
Physical inactivity								
0	10124	1,320	1.00 (reference)	459	1.00 (reference)	542	1.00 (reference)	0.13 (0.09, 0.18)
1	49623	3,632	1.20 (1.12, 1.28)	1,178	1.24 (1.11, 1.39)	1,580	1.14 (1.02, 1.26)	

N: number;

HR: adjusted for age group, education, income per person, occupation, history of hypertension, diabetes mellitus, coronary heart disease, and stroke and other individual lifestyle factor;

CI: confidence interval;

PAR: population attributable risk;

0: Not at risk;

1: At risk.

### Impact of Lifestyle Risk Index

The lifestyle index was related to increased risks of all-cause, CVD, and cancer mortality (all p values for trend < 0.001). Age-adjusted and multivariable adjusted HRs and 95% CIs are presented in Table 4. For all-cause mortality, compared to men with zero point, HRs (95%CI) for those with one, two, three, four points were 1.25 (95%CI: 1.11, 1.40), 1.69 (95%CI: 1.51, 1.89), 1.98 (95%CI: 1.76, 2.23) and 2.92 (95%CI: 2.53, 3.38) accordingly. Similar patterns were observed for CVD and cancer mortality. The PARs for these with four risk behaviors were 0.41 (95%CI: 0.33, 0.48) of all-cause mortality, 0.40 (95%CI: 0.27, 0.51) of CVD mortality and 0.38 (95%CI: 0.26, 0.49) of cancer mortality, accordingly.

**Table 4:** Hazard ratios for all-cause, cardiovascular, and cancer mortality associated to lifestyle risk index,the Shanghai Men’s Health Study (2002–2013, n = 59,747).

Lifestyle index	0	1	2	3	4
Total-mortality					
N Deaths	393	1,205	1,766	1,187	401
Model 1	1.00 (reference)	1.26 (1.12, 1.41)	1.79 (1.61, 2.00)	2.2 (1.96, 2.48)	3.37 (2.93, 3.89)
Model 2	1.00 (reference)	1.25 (1.11, 1.40)	1.69 (1.51, 1.89)	1.98 (1.76, 2.23)	2.92 (2.53, 3.38)
Disease history *					
No	1.00 (reference)	1.13 (0.93, 1.37)	1.41 (1.17, 1.70)	1.77 (1.45, 2.15)	2.29 (1.82, 2.89)
Yes	1.00 (reference)	1.28 (1.11, 1.48)	1.90 (1.66, 2.19)	2.10 (1.81, 2.44)	3.39 (2.81, 4.09)
Age*					
< 65	1.00 (reference)	0.92 (0.70, 1.20)	0.94 (0.72, 1.21)	1.13 (0.87, 1.46)	1.62 (1.23, 2.14)
> = 65	1.00 (reference)	1.23 (1.08, 1.40)	1.77 (1.57, 2.01)	1.96 (1.71, 2.24)	2.67 (2.21, 3.24)
CVD mortality					
N Deaths	135	436	566	377	123
Model 1	1.00 (reference)	1.37 (1.13, 1.67)	1.83 (1.51, 2.21)	2.28 (1.87, 2.79)	3.46 (2.70, 4.44)
Model 2	1.00 (reference)	1.39 (1.14, 1.68)	1.79 (1.48, 2.16)	2.17 (1.77, 2.65)	3.15 (2.44, 4.05)
Disease history *					
No	1.00 (reference)	1.33 (0.87, 2.03)	1.49 (0.98, 2.27)	1.79 (1.16, 2.76)	2.56 (1.54, 4.23)
Yes	1.00 (reference)	1.37 (1.10, 1.70)	1.91 (1.54, 2.36)	2.32 (1.84, 2.92)	3.29 (2.44, 4.42)
Age *					
< 65	1.00 (reference)	1.81 (0.94, 3.47)	1.85 (0.98, 3.50)	2.68 (1.41, 5.09)	3.75 (1.93, 7.31)
> = 65	1.00 (reference)	1.30 (1.06, 1.60)	1.74 (1.42, 2.13)	1.77 (1.41, 2.23)	2.30 (1.64, 3.22)
Cancer mortality					
N Deaths	151	464	791	522	194
Model 1	1.00 (reference)	1.2 (1.00, 1.45)	1.91 (1.60, 2.29)	2.27 (1.89, 2.73)	3.76 (3.02, 4.67)
Model 2	1.00 (reference)	1.18 (0.98, 1.41)	1.78 (1.49, 2.13)	2.01 (1.66, 2.42)	3.18 (2.55, 3.97)
Disease history *					
No	1.00 (reference)	1.08 (0.82, 1.42)	1.60 (1.23, 2.08)	1.85 (1.41, 2.43)	2.41 (1.75, 3.33)
Yes	1.00 (reference)	1.25 (0.97, 1.60)	1.94 (1.53, 2.47)	2.09 (1.61, 2.72)	4.34 (3.20, 5.89)
Age *					
< 65	1.00 (reference)	0.86 (0.58, 1.26)	1.02 (0.70, 1.48)	1.07 (0.73, 1.56)	1.61 (1.07, 2.42)
> = 65	1.00 (reference)	1.17 (0.95, 1.45)	1.85 (1.51, 2.27)	2.12 (1.70, 2.65)	3.35 (2.50, 4.48)

N: number;

Disease history: Self-reported hypertension, diabetes mellitus, coronary heart disease, and stroke.

Model 1: Adjusted for age group;

Model 2: Adjusted for age group, education, income per person, occupation, history of hypertension, diabetes mellitus, coronary heart disease, and stroke;

All P values for trend < 0.001;

*Model 2 was used to calculate the hazard ratio.

In stratified analysis by age group, risks of all-cause and cancer mortality with the lifestyle index in those 65 years old and over were significantly higher than in younger men. However, for CVD mortality, the risk was higher in younger men than in those 65 years old and over. When stratified by history of chronic diseases, the association between the index score and mortality risk in the group of prevalent chronic diseases patients was more pronounced than that in the group without chronic diseases. In analysis excluding deaths in the first two years, the magnitudes of associations were almost unchanged (Supplementary Table S3). And results were also not changed remarkably when further adjusted for body mass index (BMI) as an additional covariate (Supplementary Table S4).

### Combinations of Risk Factors

The distribution of all possible lifestyle combinations and their associations with risks of all-cause and cause-specific mortality are shown in Table 5. Among all the 16 mutually exclusive combinations, more than 90% participants belonged to the 8 most common combinations. Among men with only one unhealthy behavior, physical inactivity accounted for the largest percentage (16.78%), and smoking (4.16%) was followed. For men with at least two risk factors, the commonly occurred combinations were smoking with physical inactivity (27.70%), smoking with unhealthy diet and physical inactivity (19.93%), unhealthy diet with physical inactivity (7.13%), and possession of all the four risk lifestyle factors (6.54%). All the sixteen combinations except three (with small sample size) were significantly related to risk of all-cause mortality. In all single occurred risk factors, smoking had the highest risk on total mortality (HR = 1.45; 95%CI: 1.24, 1.68) and cancer mortality (HR = 1.63; 95%CI: 1.29, 2.06). Heavy alcohol use had the highest risk on CVD mortality (HR=1.83; 95% CI: 0.99-3.39). Among all combinations, possession of all the four risk factors simultaneously was most strongly associated with all-cause mortality (HR = 2.93, 95%CI: 2.54, 3.39) and cause specific mortality (CVD: HR = 3.16, 95%CI: 2.45, 4.07; cancer: HR = 3.22, 95%CI: 2.58, 4.02). Other combinations with relatively strong associations mostly were smoking with two of the other three risk behaviors, such as smoking with heavy alcohol use and unhealthy diet (total: HR = 2.24, 95%CI: 1.76, 2.84; CVD: HR = 2.16, 95%CI: 1.41, 3.32; cancer: HR = 2.70, 95%CI: 1.91, 3.83); smoking with heavy alcohol use and physical inactivity (total: HR = 2.12, 95%CI: 1.77, 2.54; CVD: HR = 2.08, 95%CI: 1.50, 2.87; cancer: HR = 2.33, 95%CI: 1.78, 3.06); smoking with unhealthy diet and physical inactivity (total: HR = 1.97, 95%CI: 1.74, 2.23; CVD: HR = 2.24, 95%CI: 1.81, 2.77; cancer: HR = 1.95, 95%CI: 1.60, 2.37). In addition, the most common combination (smoking with physical inactivity) also had relative strong association with all-cause (HR = 1.70; 95%CI: 1.51, 1.93), CVD (HR = 1.79; 95%CI: 1.44, 2.21) and cancer mortality (HR = 1.90; 95%CI: 1.57, 2.30).

**Table 5:** Prevalence of all 16 combinations of lifestyle risk behaviors and adjusted hazard ratios for their associations with risks of all-cause, cardiovascular, and cancer mortality, the Shanghai Men’s Health Study (2002–2013, n = 59,747).

Risk factors	All-Cause HR (95% CI)	CVD HR (95% CI)	Cancer HR (95% CI)	Cohort N	Percentage (%)
None	1.00 (reference)	1.00 (reference)	1.00 (reference)	3,626	6.07
Smoking	1.45 (1.24, 1.68)	1.59 (1.23, 2.06)	1.63 (1.29, 2.06)	2,488	4.16
Alcohol	1.34 (0.88, 2.02)	1.83 (0.99, 3.39)	1.31 (0.67, 2.57)	143	0.24
Diet	1.2 (1.02, 1.42)	1.47 (1.13, 1.93)	1.04 (0.78, 1.38)	1,419	2.38
PA	1.19 (1.05, 1.35)	1.27 (1.03, 1.58)	1.06 (0.87, 1.30)	10,027	16.78
Smoking + alcohol	1.94 (1.49, 2.54)	1.77 (1.09, 2.86)	2.55 (1.75, 3.73)	380	0.64
Smoking + diet	1.75 (1.49, 2.06)	1.69 (1.27, 2.26)	1.83 (1.42, 2.35)	1,513	2.53
Smoking + PA	1.70 (1.51, 1.93)	1.79 (1.44, 2.21)	1.90 (1.57, 2.30)	16,549	27.70
Alcohol + diet	1.45 (0.93, 2.28)	1.29 (0.57, 2.93)	1.56 (0.77, 3.19)	101	0.17
Alcohol + PA	1.05 (0.71, 1.55)	1.49 (0.84, 2.63)	0.92 (0.47, 1.81)	320	0.54
Diet + PA	1.68 (1.48, 1.92)	1.89 (1.51, 2.37)	1.55 (1.24, 1.92)	4,262	7.13
Smoking + alcohol + diet	2.24 (1.76, 2.84)	2.16 (1.41, 3.32)	2.70 (1.91, 3.83)	454	0.76
Smoking + alcohol + PA	2.12 (1.77, 2.54)	2.08 (1.50, 2.87)	2.33 (1.78, 3.06)	2,316	3.88
Smoking + diet + PA	1.97 (1.74, 2.23)	2.24 (1.81, 2.77)	1.95 (1.60, 2.37)	11,909	19.93
Alcohol + diet + PA	1.46 (1.04, 2.05)	1.38 (0.75, 2.56)	1.35 (0.76, 2.38)	333	0.56
Smoking + alcohol + diet + PA	2.93 (2.54, 3.39)	3.16 (2.45, 4.07)	3.22 (2.58, 4.02)	3,907	6.54

HR: Hazard ratioe. Adjusted for age group, education, income per person, occupation, history of hypertension, diabetes mellitus, coronary heart disease, and stroke.

CI: Confidence interval; N: number; PA: physical activity.

0: Not at risk; 1: At risk.

## Discussion

In the current analysis, we found increasing risks on all-cause and cause-specific mortality as the number of risk lifestyle factors added. Compared to men with none unhealthy behavior, the hazard ratios rose from 1.25 to 2.92 for individuals with one to four risk lifestyle factors for all-cause mortality, and 1.39 to 3.15 for CVD mortality, and 1.18 to 3.18 for cancer mortality, respectively. For the four behavior factors understudy, only 6.07% men had none unhealthy lifestyle factors in our study population. The population attributable risks to those four risk factors were 0.41 for total deaths, 0.40 for CVD deaths and 0.38 for cancer deaths.

A number of epidemiological studies have investigated the combined impact of lifestyle factors on health outcome. Though all studies reported an inverse relationship between number of healthy behaviors and risk of mortality, the effect magnitudes varied much. A recent meta-analysis reported a 66% reduction of all-cause mortality by adherence to at least four healthy lifestyle factors, while substantial heterogeneity was found between study populations (I^2^ = 94%)^22^.

For comparing the highest- with the lowest- healthy lifestyle index, the relative risk estimates ranged from 0.20 (95%CI: 0.15, 0.28)^18^ to 0.58 (95%CI: 0.52, 0.64)^11^. The variation in the number and types of lifestyle factors may be one of the main reasons for such heterogeneity in results. Among previous studies, most studies chose non-smoking, limited alcohol use, healthy diet, and active physical activity in the construction of the index^7, 14, 16, 26–28^, and some also included anthropometric measures as a factor^8^. In addition, a few studies added some non-traditional factors such as sedentary time, sleep and even social networking in the creation of the index score^18, 19, 21, 29^. In the current study, our purpose was to investigate the impact of these traditional lifestyle factors independent of anthropometric measures on mortality outcome. Another important explanation for the heterogeneity was the difference in the definition of healthy behavior. Specifically, for smoking, some studies took never-smoking as low-risk^8, 16, 19, 30^, while others used never-smoking and long-term non-smoking as healthy behavior^12, 18, 20, 28, 31^. In several studies, those that took light to moderate alcohol were categorized in low-risk group^7, 8, 10, 16, 19, 32^, but in other researches, those with none or light to moderate alcohol intake were all assigned to the healthy group^14, 20, 29^. As to physical activity, studies have considered the duration^7, 8, 11, 12, 14, 17, 19, 29, 31^ or frequency^16, 20, 27, 32^ of moderate to vigorous physical activity per week separately. Moreover, methods evaluating diet differed. Mediterranean diet scores^12, 31^, intake of fruit and vegetables^14, 17, 27^, index based on dietary guidelines^8, 16, 18, 29^, or serum vitamin C level^7, 26^ were applied in various studies. In our analysis, these lifestyle factors were defined and categorized according to recommendations of public health^33–35^. For diet, a Chinese Food Pagoda (CHFP) score was used as it was more suitable for Chinese population and was easy to understand for the public.

Due to the great heterogeneity in the distribution of lifestyle factors between men and women in Shanghai, the current study focused on men. Actually, in a previous study, based on another five lifestyle-related factors (normal weight, lower waist-hip ratio, daily exercise, never exposed to spouse’s smoking, higher daily fruit and vegetable intake), we investigated the combined impact of lifestyle in the Shanghai Women’s Health Study^17^. Compared to women with a score of zero, hazard ratios (95% confidence intervals) for women with four to five factors were 0.57 (0.44–0.74) for total mortality, 0.29 (0.16–0.54) for CVD mortality, and 0.76 (0.54–1.06) for cancer mortality. Only a few studies have reported the association between combined lifestyle and mortality in Asian women. A Korean cohort study found that compared to those having none or only one risk factor, the relative risks in women with a combination of four lifestyle factors was 2.00-fold for cancer mortality, 2.17-fold for non-cancer mortality, and 2.09-fold for all-cause mortality^20^. The Japan Collaborative Cohort Study reported that age-adjusted HR (95% CIs) of all-cause mortality for the group with 6 healthy lifestyle factors was 0.49 (0.39–0.60) among women compared with the group with 0–2 healthy lifestyle factors^21^. Despite of differences in number, type and the definition of lifestyle behaviors, results from these studies suggested that the joint lifestyle not only have an impact on men but also on women in Asia.

For the estimation of PAR, the heterogeneity of the results was also considerable, ranging from 19%^21^ to 60%^28^. PAR was influenced not only by the risks of the specific lifestyle factors but also by the prevalence of those factors in study population. On the one hand, exposure pattern in the SMHS was very different from that in western cohort studies. For example, smoking prevalence was as high as 66% in our population compared to 50% in UK^14^, 44% in US^16^, and 7% in Australia cohort studies^29^. Besides, East Asians tend to be physically inactive relative to their counterparts in western countries^36, 37^. Only 17% participants reached the WHO recommendation of physical activity level in the SMHS, while the percentage was 73% in UK^7^ and 77% in Australia^29^, 40% in US^16^ cohort studies. Therefore, calculation of population specific PAR was critically essential for policy making in this area. On the other hand, results in the SMHS are of significance for men in other cities of China and in other Asian countries who have similar ethnicity and cultural factors. One of the few studies in Asian men found that the population attributable risk for all-cause mortality to the four risk factors combined was 45% for Korean men^20^. The Japan Collaborative Cohort Study indicated 49% of deaths among men could be prevented if all men adopted all the following 6 healthy lifestyle factors: not currently smoking, not heavily drinking, walking 1 h or more per day, sleeping 6.5 to 7.4 h per day, eating green-leafy vegetables almost daily and BMI between 18.5 and 24.9. All these findings supported our conclusion that healthy lifestyle was indispensable in reducing the morality burden in Asian men.

Compared to previous studies, we additionally analyzed the impact of all the possible factor combinations on risks of total and cause-specific mortality. Among all the 16 combinations, we found over 75% participants have at least two risk behaviors. As the most common single risk lifestyle factor, HR of physical inactivity for the association with total mortality on its own was only 1.19. However, when it co-existed with unhealthy diet, HR augmented apparently (HR for all-cause mortality: 1.68) though the single effect of unhealthy diet on all-cause mortality was also only 1.20. This finding may indicate that men with both physical inactivity and poor diet may be at higher risk of deaths. Similar joint effect was also reported in a previous study on chronic diseases which found a larger risk reduction in combination of physical activity and healthy diet compared to that of the single factor^38^.

For the influence of each individual behavior, smoking and heavy alcohol use had stronger association with risk of total and cancer mortality. For the whole population, however, smoking and physical inactivity had the highest PAR because of their high prevalence in our cohort, even though the HR was only 1.14 for physical inactivity. Therefore, in terms of intervention, emphasis should be put on encouraging smoking cessation and taking exercise more.

There are some strengths in our study. Because of the high response rate and follow-up rate, our results were less likely to be influenced by selection bias. We adjusted for most possible confounders making the analysis less likely to be influenced by confounding bias. Furthermore, all variables were measured by trained staffs with standard proposal and the outcome information was proved almost 100% complete. Finally, the relatively large sample size allowed us to explore the effect of all possible lifestyle combinations and conduct sub-group analysis. In addition, the association stays robust in various sensitivity analyses, which proved the validation of our results.

Some limitations should be considered. First, all lifestyle behaviors were self-reported, which may introduce measurement errors especially for diet and physical activity. However, both the food frequency questionnaire and physical activity questionnaire have been validated and shown good reliability^39, 40^. Second, all variables used the single measurement at baseline. Though we assumed lifestyle remained stable as time passed and did not found the violation of proportional hazards assumption statistically, we still could not exclude the possibility of some changes in lifestyle factors. Third, as the long latency of many chronic diseases, reverse causation bias may influence our results. Finally, dichotomizations of variables might reduce statistical efficiency. However, our primary aim was to evaluate the risk of mortality using a simple method which could be easily applied by general population. Besides, the result did not change largely in our supplement analyses by reconstructing lifestyle index using multi-classification of individual lifestyle factors.

In conclusion, our findings suggested that combined risk lifestyle behaviors had substantial impact on total and cause-specific mortality. About 41% of deaths in our cohort participants were attributable to not adhering to these four healthy behaviors. It is worth noting that only 6% men followed all these four healthy lifestyle factors in the population. This sends a strong message to policymakers to make adopting healthy lifestyle a top public health priority in reducing the burden of chronic disease related morality.

## Methods

### Study Population

Participants included in the current analysis are from the SMHS, a population based prospective cohort conducted in eight typical neighborhood communities of urban Shanghai. Details about the SMHS have been previously reported elsewhere^23, 41^. Briefly, of 82,043 eligible permanent male residents aged 40 to 74 years with no history of cancer, 61,480 were recruited from March 2002 through June 2006, with a response rate of 74%. Information concerning demographics, medical history, family history of cancer and such lifestyle factors as tobacco use, alcohol drinking, usual diet and physical activity was collected with an in-person interview. Anthropometry data like height, weight, waist and hip circumstance were measured by trained staffs using standard methods. Both the food frequency and physical activity questionnaires have been validated in the cohort^39, 40^. The validity of the food frequency questionnaire was evaluated by comparing nutrient and food group intake levels from the second food frequency questionnaire and the multiple 24-HDR questionnaires, with correlation coefficients ranging from 0.35 to 0.72 for food groups^39^. The validity of the physical activity questionnaire was evaluated by comparing with criterion measures: 1-year averages of 7-day physical activity recall and physical activity log. Correlations between the physical activity questionnaire and criterion measures of adult exercise were 0.45 (7-day physical activity recall) and 0.51 (physical activity log)^40^.

The first two in-person follow-up surveys were finished to obtain interim health history with follow-up rates of 97.6% and 93.7% in 2004–2008 and 2008–2011. In addition, we conducted annual record linkages to the Shanghai Cancer Registry and the Shanghai Vital Statistics to identify additional cancer cases and vital status.

### Assessment of Lifestyle Risk Factors

Baseline information on lifestyle factors was applied to the current analysis. For smoking, participants were asked the smoking status (never, former, or current smoker); age of starting smoking for smokers; age of smoking cessation for former smokers. Alcohol consumption was estimated according to the frequency of drinking specific alcoholic beverages. We then calculated alcohol intake based on the ethanol content of each type of beverage (10 g ethanol/100 g rice wine; 4 g ethanol/100 g beer; 40 g ethanol/100 g liquor; 12 g ethanol/100 g grape wine). Finally, we expressed the intake of alcohol in units of “drink” which equals to 14.18 grams of ethanol. The physical activity was measured using physical activity questionnaire^40^. Individuals were defined as exercisers if they had taken exercise at least once a week for more than three months continuously during the past five years. Inquiries for exercisers included types, duration for one week and years of participation for up to three activities. We first transformed the information into standard metabolic equivalents as MET-hours/day, and then calculated exercise time by taking 1 MET-hours equivalent to about 15 minutes of moderate-intensity activities^42^.

Dietary behavior was assessed using a CHFP score as higher CHFP score was proved to be related to lower mortality^43^. The CHFP score was constructed according to the Chinese dietary guidelines using methods of creating US Eating Index 2005^44^. Briefly, the following ten food components were included in the CHFP score: grains, vegetables, fruits, dairy, beans, meat and poultry, fish and shrimp, eggs, fats and oils as well as salt. We first used the density method to adjust dietary intakes for energy intake to 2000 kcal/d^35^. Then, we defined maximum points and zero point according to recommended intakes in the Food Pagoda^35^. Scores for dietary intakes between minimum and maximum amounts were calculated proportionately. The final CHFP score ranging from 0 to 45 was the sum of each component scores. Details on methods to build the CHFP score have been described elsewhere^43^.

For individual lifestyle factor, we coded as one for a person who was at risk and as zero for those who was not at risk. Specific definitions of “at risk” or “not at risk” for these lifestyle factors were based on public health recommendations and guidelines (Table 1)^33–35^. A lifestyle risk index was calculated for each man by summing the codes of the four factors (total score range 0–4).

### Assessment of Death

Information of vital status and causes of deaths was collected through a combination of active follow-up and annual record linkage to the Shanghai Vital Statistics. The current analysis used death data updated to December 31, 2013. Death causes were coded using the International Classification of Diseases, the Ninth (ICD-9) in which cardiovascular diseases (CVD) were coded as 390–459 and cancers as 140–208^45^.

### Statistical Analysis

Among 61,480 men who completed the baseline questionnaires, we excluded 256 participants with extreme energy intake (<800 kcal or > 4,000 kcal per day). We also excluded men with missing data on individual lifestyle factors (smoking: 1; alcohol use: 311) or covariates (n = 1,159) and men with missing follow-up information (n = 6). The remaining 59,747 participants were included in the current analysis.

We described the distribution of baseline characteristics according to the number of unhealthy lifestyle factors. Continuous variables were presented with mean (standard deviation) and categorical variables with frequency (percentage).

Cox proportional hazards models were used to assess risks of all-cause and cause-specific mortality associated with each individual lifestyle factors as well as the combined lifestyle index. Follow-up time was taken as time scale in these models. Survival time was calculated from the date of baseline interview to the date of death or December 31, 2013, whichever came first. Age-adjusted and multivariable-adjusted models were constructed separately. Multivariable model adjusted for almost all possible confounders: age group at baseline (40–44, 45–49, 50–54, 55–59, 60–64, 65–69, 70–74), occupation (professional, clerical and manual workers), education (none/elementary, junior high school, high school, above high school), income/person (<500 CNY, 500–999 CNY,1,000–1,999 CNY, >  = 2,000 CNY), and history of hypertension, coronary heart disease, diabetes mellitus and stroke. The linear trends were evaluated by taking the index score as a continuous variable in models. In analyses of individual lifestyle factors, besides adjustments above, we additionally mutually adjusted for four lifestyle factors (smoking, alcohol use, physical inactivity and dietary behavior). Proportional hazards assumption was evaluated by testing the significance of interaction terms between lifestyle index, all covariates and follow-up time in main analyses and sensitivity analyses, and no evidence of departure from proportional hazards was detected.

To test the possibility of modification, we made stratified analyses by age (<65 vs. >  = 65) and history of chronic diseases (hypertension, coronary heart disease, diabetes mellitus and stroke). Sensitivity analyses was conducted by excluding deaths in the first two years of follow-up and we further adjusted for BMI in the multivariable adjusted model as BMI may be a potential confounder. Finally, to assess the association between specific lifestyle patterns with all-cause and cause-specific mortality, we created sixteen variables for all possible combinations of these four lifestyle factors. Percentages and multivariable-adjusted hazard ratios (HRs) for all combinations were provided. Additionally, we conducted detailed analyses by recalculating the lifestyle risk index score using multi-categories of individual lifestyle factors (see Supplementary Table S1 and Supplementary Table S2). Multi-categories analyses did not change the conclusion that the relative risk of mortality increased as the number of risk lifestyle factors added. As the dichotomization method was more easily to be understood and applied, lifestyle factors were categorized into two groups in main analyses.

Multivariable adjusted PARs and 95% CIs were calculated by summing specific PAR for each lifestyle index compared to index of zero^46, 47^. PAR can be explained as the proportion of deaths that could be avoided if all participants had zero risk lifestyle factors^48^. PARs for individual lifestyle factor was also calculated. The following formula was applied to calculate PAR:$\sum _{i=0}^{k}p{d}_{i}(R{R}_{i}-1)/R{R}_{i}\times 100$, where *pd*_*i*_ represents the proportion of cases in the *i* ^*th*^ exposure; *RR*_*i*_ means the relative risk by comparing men in the *i* ^*th*^ exposure group to those in the reference group. PAR estimates assumed that the observed associations were causal^46^.

### Human subjects review

The study was approved by the Institutional Review Boards of Vanderbilt University and the Shanghai Cancer Institute and conducted in accordance with The Declaration of Helsinki Principles. Informed consent was obtained from all participants.

## Electronic supplementary material

Supplemental Tables S1–4
