# Supplementary material for: Combined Impact of Known Lifestyle Factors on Total and Cause-Specific Mortality among Chinese Men: A Prospective Cohort Study
Source: Sci Rep. 2017 Jul 13;7:5293. doi: 10.1038/s41598-017-05079-5 (PMC5509739; doi:10.1038/s41598-017-05079-5)
Supplement: Supplementary file 1 — Supplemental Tables S1–4 [file 41598_2017_5079_MOESM1_ESM.pdf]

Combined Impact of Known Lifestyle Factors on Total and Cause-Specific Mortality among Chinese Men: A Prospective Cohort Study

Qing-Li Zhang, Long-Gang Zhao, Wei Zhang, Hong-Lan Li, Jing Gao, Li-Hua Han, Wei Zheng, Xiao-Ou Shu, Yong-Bing Xiang

Supplementary Table S1: Scoring of risk factors in lifestyle risk index, the Shanghai Men's Health Study (2002-2013, n = 59747)

Health Behavior	Classification	Scoring Classification
Smoking[a]	Never	0
	Former	1
	Current light	2
	Current heavy	3
Alcohol use[b]	Nondrinker	1
	Moderate	0
	Heavy	2
Dietary behavior	Tertile1	2
	Tertile2	1
	Tertile3	0
Physical activity	No activity	2
	0 to 150min/week	1
	>=150min/week	0

[a] Light current smoking is defined as smoking for <20 years or <20 cigarettes/day; Heavy current smoking is defined as smoking for>=20 years and >=20 cigarettes/day.

[b] Moderate alcohol intake is defined as >0 but <=14 drinks/week; Heavy alcohol intake is defined as >14 drinks/week.

Supplementary Table S2: Hazard ratios for all-cause, cardiovascular, and cancer mortality associated to lifestyle risk index, the Shanghai Men's Health Study (2002-2013, n = 59747)

Lifestyle score	0-2	3-4	5-6	7-9	P for trend
Total-mortality					
N Deaths	714	1580	1641	1017	
Model 1	1.00 (reference)	1.43 (1.31,1.56)	1.86 (1.70,2.03)	2.46 (2.23,2.72)	<0.01
Model 2	1.00 (reference)	1.36 (1.24,1.48)	1.68 (1.54,1.84)	2.2 (1.99,2.44)	<0.01
CVD mortality					
N Deaths	254	546	529	308	
Model 1	1.00 (reference)	1.44 (1.24,1.67)	1.84 (1.58,2.14)	2.4 (2.02,2.85)	<0.01
Model 2	1.00 (reference)	1.37 (1.18,1.60)	1.72 (1.47,2.00)	2.33 (1.95,2.78)	<0.01
Cancer mortality					
N Deaths	275	650	696	501	
Model 1	1.00 (reference)	1.47 (1.28,1.70)	1.89 (1.64,2.18)	2.81 (2.41,3.27)	<0.01
Model 2	1.00 (reference)	1.41 (1.22,1.62)	1.71 (1.48,1.98)	2.44 (2.09,2.86)	<0.01

N: number;

Model 1: Adjusted for age group;

Model 2: Adjusted for age group, education, income per person, occupation, history of hypertension, diabetes mellitus, coronary heart disease, and stroke.

Supplementary Table S3: Hazard ratios for all-cause, cardiovascular, and cancer mortality associated to lifestyle risk index, the Shanghai Men's Health Study (2002-2013, n = 59747) , sensitivity analysis 1

Lifestyle index	0	1	2	3	4	P for trend
Total-mortality						
N Deaths	349	1058	1555	1023	345	.
Model 1	1.00 (reference)	1.24 (1.10,1.40)	1.79 (1.59,2.02)	2.16 (1.91,2.44)	3.32 (2.85,3.87)	<0.01
Model 2	1.00 (reference)	1.23 (1.09,1.39)	1.69 (1.50,1.91)	1.95 (1.72,2.22)	2.90 (2.48,3.38)	<0.01
CVD mortality						
N Deaths	117	374	495	326	106	
Model 1	1.00 (reference)	1.37 (1.11,1.68)	1.87 (1.52,2.29)	2.31 (1.86,2.87)	3.52 (2.70,4.60)	<0.01
Model 2	1.00 (reference)	1.38 (1.12,1.70)	1.83 (1.49,2.24)	2.19 (1.76,2.72)	3.21 (2.45,4.22)	<0.01
Cancer mortality						
N Deaths	136	426	703	451	173	
Model 1	1.00 (reference)	1.22 (1.01,1.48)	1.88 (1.56,2.26)	2.16 (1.78,2.63)	3.70 (2.94,4.65)	<0.01
Model 2	1.00 (reference)	1.19 (0.98,1.45)	1.75 (1.45,2.11)	1.92 (1.58,2.35)	3.16 (2.50,3.99)	<0.01

Sensitivity analysis 1:Excluding deaths in the first two years;

N: number;

Disease history: Self-reported hypertension, diabetes mellitus, coronary heart disease, and stroke;

Model 1: Adjusted for age group;

Model 2: Adjusted for age group, education, income per person, occupation, history of hypertension, diabetes mellitus, coronary heart disease, and stroke.

Supplementary Table S4: Hazard ratios for all-cause, cardiovascular, and cancer mortality associated to lifestyle risk index, the Shanghai Men's Health Study (2002-2013, n = 59747), sensitivity analysis 2

Lifestyle index	0	1	2	3	4	P for trend
Total-mortality						
N Deaths	393	1205	1766	1187	401	.
HR (95%CI)	1.00 (reference)	1.24 (1.11,1.39)	1.67 (1.49,1.87)	1.94 (1.72,2.18)	2.85 (2.47,3.29)	<0.01
CVD mortality						
N Deaths	135	436	566	377	123	
HR (95%CI)	1.00 (reference)	1.38 (1.14,1.68)	1.78 (1.47,2.15)	2.14 (1.75,2.62)	3.12 (2.42,4.02)	<0.01
Cancer mortality						
N Deaths	151	464	791	522	194	
HR (95%CI)	1.00 (reference)	1.17 (0.98,1.41)	1.76 (1.48,2.11)	1.97 (1.63,2.38)	3.12 (2.50,3.89)	<0.01

Sensitivity analysis 2: Further adjusted for body mass index (BMI) as an additional covariate;

N: number;

Disease history: Self-reported hypertension, diabetes mellitus, coronary heart disease, and stroke;

Adjusted for age group, education, income per person, occupation, history of hypertension, diabetes mellitus, coronary heart disease, and stroke.
